# STS-AT: A Structured Tensor Flow Adversarial Training Framework for Robust Intrusion Detection

**DOI:** 10.3390/s26020536

**Published:** 2026-01-13

**Authors:** Juntong Zhu, Zhihao Chen, Rong Cong, Hongyu Sun, Yanhua Dong

**Affiliations:** 1 Computer Science and Technology, School of Mathematics and Computer Science, Jilin Normal University, Siping 136000, China; zjt@malis.jlnu.edu.cn (J.Z.); cicy816@mails.jlnu.edu.cn (R.C.); 2 Asiainfo Security Technologies Co., Ltd., Nanjing 210000, China; williamschen2021@outlook.com

**Keywords:** network intrusion detection, structured tensor, raw traffic, adversarial training, robustness

## Abstract

**Highlights:**

**What are the main findings?**

- We propose a Structured Tensor Streaming with Adversarial Training (STS-AT) framework that achieves over 99% accuracy in normal traffic classification and maintains over 96.8% accuracy under various adversarial attacks (FGSM, PGD, and DeepFool).
- The multi-strategy adversarial training method significantly enhances model robustness, raising defense accuracy from as low as 24.4% (under FGSM attack on undefended models) to above 97.1%.

**What are the implications of the main findings?**

- We provide a novel methodology that integrates structured raw traffic representation with efficient adversarial training, offering a systematic solution to the dual challenges of feature information loss and model vulnerability in intrusion detection.
- The efficient training strategy reduces total training time by approximately 67.6% compared to sequential training methods, demonstrating strong potential for practical deployment in real-world network environments.

**Abstract:**

Network intrusion detection is a key technology for ensuring cybersecurity. However, current methods face two major challenges: reliance on manual feature engineering, which leads to the loss of discriminative information, and the vulnerability of deep learning models to adversarial sample attacks. To address these issues, this paper proposes STS-AT, a novel network intrusion detection method that integrates structured tensors with adversarial training. The method consists of three core components: first, structured tensor encoding, which fully converts raw hexadecimal traffic into a numerical representation; second, a hierarchical deep learning model that combines CNN and LSTM networks to simultaneously learn spatial and temporal features of the traffic; third, a multi-strategy adversarial training method that enhances model robustness by adaptively adjusting the mix of adversarial samples in different training phases. Experiments on the CICIDS2017 dataset show that the proposed method achieves an accuracy of 99.6% in normal traffic classification, significantly outperforming classical machine learning baselines such as Random Forest (93.1%) and Support Vector Machine (84.7%). Crucially, under various adversarial attacks (FGSM, PGD, and DeepFool), the accuracy of an undefended model drops to as low as 24.4%, whereas after multi-strategy adversarial training, the defense accuracy rises above 96.8%. Meanwhile, the total training time is reduced by approximately 67.6%. These results verify that structured tensor encoding effectively preserves original traffic information, the hierarchical model achieves comprehensive feature learning, and multi-strategy adversarial training significantly improves training efficiency while ensuring robust defense effectiveness.

## 1. Introduction

Over the past decade, the Internet has developed rapidly in terms of scale, protocol complexity, and application diversity [1]. Network attacks have become increasingly frequent, posing a serious threat to infrastructure, enterprise operations, and personal privacy [2]. As a key link in network security, the evolution of network intrusion detection technology has shown a trend from manual experience to data-driven development. However, current research methods still face two core challenges.

**Figure 1:**
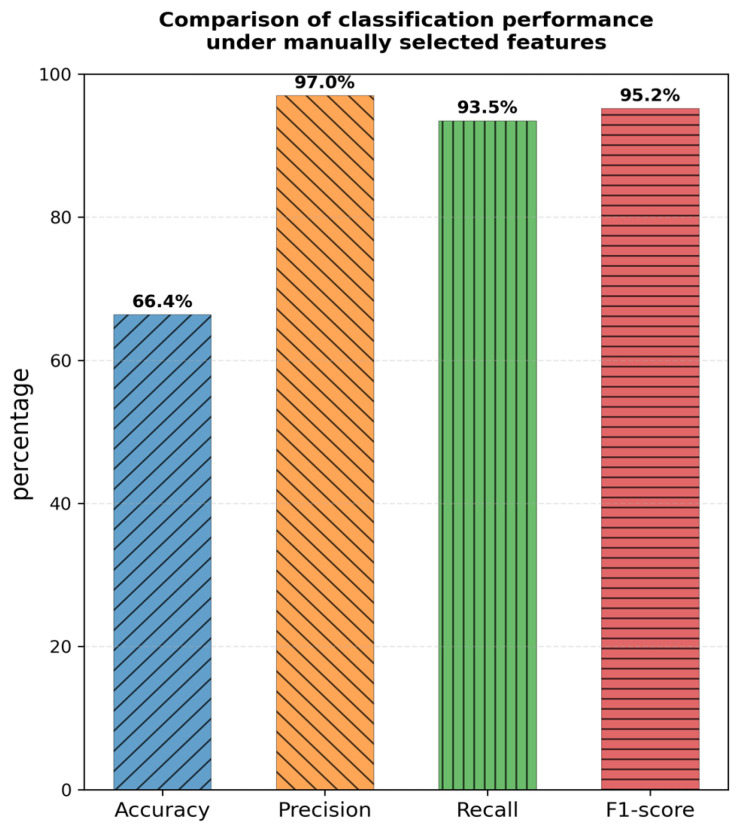
Comparison of classification performance under manually selected features.

Traditional detection methods rely on artificial feature engineering, which makes it difficult to comprehensively capture complex and changing attack patterns. Early methods based on port and protocol matching were simple to implement but easily bypassed [3]. Although payload-based deep packet inspection technology can accurately identify known threats, it cannot cope with encrypted traffic and zero-day attacks [4]. Machine learning methods such as support vector machines and random forests still require experts to construct feature systems, a process that not only consumes manpower but also leads to the loss of key discriminative information due to feature selection [5]. Experiments show that the accuracy of the model based on 32 commonly used statistical features is only 66.4%, as shown in Figure 1, a result that is far from meeting practical requirements.

**Figure 2:**
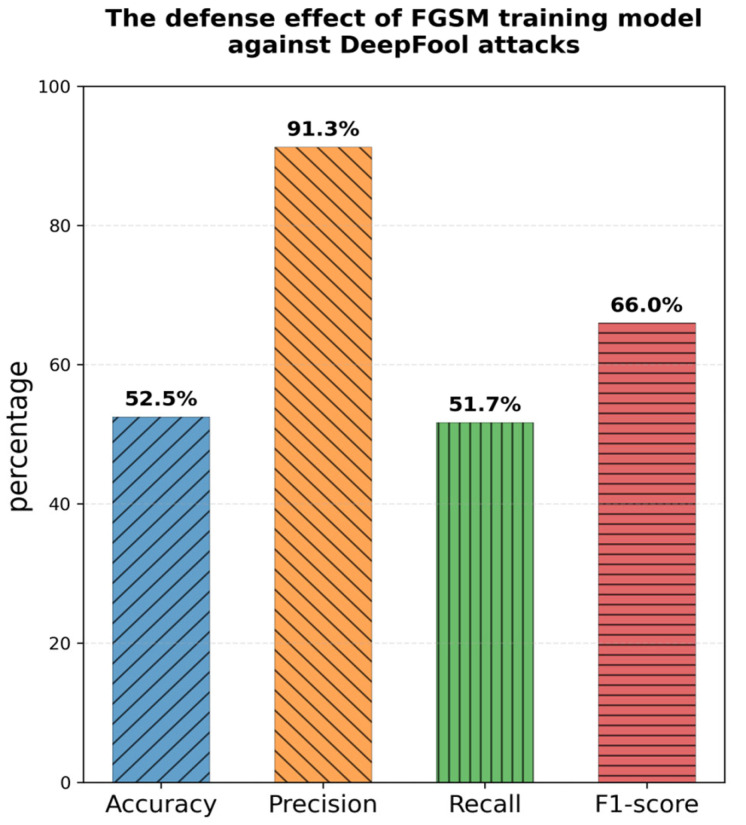
Defense effect of the FGSM training model against DeepFool attacks.

Deep learning-based detection models exhibit significant vulnerability to adversarial samples. Research has shown that attackers can generate adversarial samples to mislead models by applying subtle, human-imperceptible perturbations to malicious traffic [6]. Typical white-box attack methods, such as FGSM [7], PGD, and DeepFool [8], can all lead to a sharp decline in model performance. Although adversarial training has proven to be an effective means of improving model robustness, traditional methods face the dual challenges of limited defense range and high computational cost. Training against a single attack type can only make the model resistant to that specific attack. The experimental results of the model trained with FGSM under a DeepFool attack are shown in Figure 2. Although integrating multiple attacks into adversarial training can improve generalization robustness, it can lead to a doubling of training time, making it difficult to widely adopt in practical deployment.

Overall, building an intrusion detection system that can learn effectively from raw traffic while maintaining robustness in adversarial environments remains an unresolved challenge. Existing work either focuses on improving feature engineering or network architecture while neglecting model adversarial robustness, or it introduces adversarial training that is often impractical due to a single attack mode or high computational costs. Therefore, a framework capable of systematically integrating efficient representation of raw data, comprehensive feature learning, and effective multi-threat adversarial defense is crucial for advancing the practical application of intrusion detection technology.

To address these challenges, this paper proposes an intrusion detection scheme that integrates structured tensors with adversarial training. The main contributions are threefold: (1) the design of a structured tensor encoding method to directly learn features from the original hexadecimal traffic, thereby avoiding information loss inherent in manual feature engineering; (2) the construction of a hierarchical deep learning model and combined CNN and LSTM networks for the comprehensive extraction of spatiotemporal features; and (3) the proposal of a multi-strategy adversarial training method that adjusts the mix of adversarial samples across stages to significantly improve training efficiency without compromising defense effectiveness.

## 2. Related Work

The technology of network intrusion detection has evolved from reliance on manual experience to data-driven intelligent analysis. The research progress in this field is mainly reflected in three aspects: detection methods, core algorithms, and adversarial attacks and defense.

### 2.1. The Evolution of Network Intrusion Detection Methods

Early research on network intrusion detection relied on port and protocol matching. Moore et al. used standard ports for protocol identification [3], and Dainotti et al. extended the application of this method in specific environments [9]. Sen et al. explored a P2P traffic classification method based on payload [10], and Wang et al. proposed an anomaly worm detection framework based on byte statistics [11]. El Maghraby et al. used deep packet inspection techniques to identify malicious messages [4].

To enhance detection capabilities, researchers introduced machine learning methods. Münz et al. applied K-means clustering to real-time intrusion recognition [12], while Ertam et al. integrated genetic algorithms and extreme learning machines to improve classification performance [13]. Li et al. used PCA and random forest to construct a multi-level feature extraction scheme [14], while Zheng Liming et al. used manual feature extraction combined with the SVDD algorithm for detection [5]. However, these methods still relied on manual feature engineering, as seen in the work of Ge Qinglin et al., who used manually specified features with decision trees for attack prediction [15].

In recent years, deep learning based end-to-end methods have become mainstream, as they can automatically learn features from raw or low-level data [16]. Vinayakumar et al. proved that deep learning has superior performance in intrusion detection [17], and Wang et al. constructed an end-to-end 1D-CNN traffic classification system [18]. To improve the performance of the model, researchers explored various network architectures. Wang et al. converted traffic data into images and used a CNN for anomaly detection [19], Yin et al. proposed an RNN-IDS model to mine traffic temporal features [20], Hwang et al. used LSTM for packet classification [21], and Nath et al. used LSTM to detect DDoS attacks [22]. Wang et al. combined a CNN and BiLSTM to fuse spatiotemporal features [19], while Ren et al. used tree-structured recurrent neural networks to improve classification efficiency [23]. For instance, Gueriani et al. designed an LSTM-CNN-Attention model for IIoT environments, achieving high-precision classification on the Edge IoTSet dataset [24] and also demonstrated the effectiveness of the CNN-LSTM model on datasets including CICIDS2017 [25]. In addition, methods based on graph neural networks (such as Transformers) have also shown potential for IoT network relationship data [26]. These studies collectively advance the detection performance of deep learning in specific scenarios. However, they predominantly focus on improving accuracy in normal environments and have not systematically addressed the inherent vulnerability of deep learning models themselves to adversarial sample attacks.

### 2.2. Research on Counterattack and Defense

With the development of deep learning algorithms, their vulnerability to small perturbations of adversarial samples has been discovered. Yuan et al. revealed the models’ vulnerability to adversarial samples [6], and Papernot et al. demonstrated the transferability of adversarial samples between different models [27]. Goodfellow et al. proposed the Fast Gradient Sign Method (FGSM) to generate adversarial samples [7], Tramèr et al. applied the projected gradient descent (PGD) method to flow data [2], and Moosavi Dezfooli et al. proposed the DeepFool method to calculate the minimum perturbation [8]. To enhance the robustness of the model, adversarial training has become one of the most mainstream defense paradigms. Madry et al. established a theoretical framework for adversarial training [28], Chen et al. reduced false positive rates through stacked ensemble adversarial training [29], Shafahi et al. studied the efficiency of adversarial training [2], and Haroon et al. evaluated the effectiveness of adversarial training in intrusion detection [30]. Traditional methods, such as single-attack adversarial training (e.g., using only FGSM), have a limited defense range. Recent research has explored more efficient frameworks, such as the AIDTF framework [31], which draws on the idea of generative adversarial networks to dynamically generate diverse attack samples through adversarial games between an adversarial generation module and a detection module to enhance the model. Another work, AdvAs-IDS [32], applies adversarial samples to incremental learning scenarios, utilizing their regularization effect to alleviate catastrophic forgetting and improve the model’s generalization ability to old attack categories. In addition, a systematic review points out that in complex scenarios, such as the Internet of Things, integrating privacy protection training paradigms such as federated learning with adversarial robustness research is becoming a cutting-edge trend [33]. These works demonstrate the effectiveness of adversarial training, but often face trade-offs in terms of training efficiency or general defense against multiple attacks.

### 2.3. Comparison Between This Work and Existing Methods

Although progress has been made, a significant challenge remains in balancing high robustness, high training efficiency, and extensive defense against diverse attacks. Many adversarial training methods incur huge computational overhead or are optimized primarily for specific attack types. The STS-AT framework proposed in this paper aims to address these challenges systematically. Compared with the existing work, our core innovation is in a structured, multi-stage, multi-strategy adversarial training method. By dynamically adjusting the mix of multiple attack samples (FGSM, PGD, and DeepFool) across different training stages, this approach endows the model with comprehensive robustness in a single training cycle, significantly outperforming baseline methods that require multiple rounds of sequential training.

## 3. Methodology of This Article

**Figure 3:**
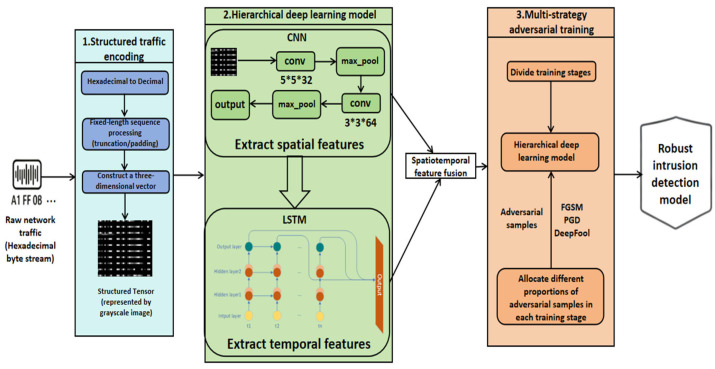
Overall flowchart of STS-AT.

This article proposes STS-AT (Structured Tensor Streaming with Adversarial Training), a network intrusion detection method that integrates structured tensors with adversarial training. The framework comprises three core components, forming a complete pipeline from raw data to robust classification. First, raw network traffic is converted into a numerical tensor via structured tensor encoding to preserve complete information. Second, a hierarchical deep learning model is designed to automatically learn spatiotemporal features from encoded data. Finally, a multi-strategy adversarial training method is adopted to enhance model robustness, enabling it to withstand various adversarial attacks. The overall workflow of the framework is illustrated in Figure 3.

### 3.1. Structured Tensor Encoding

Artificial feature engineering relies on expert knowledge to construct features from raw data, which can easily introduce numerous redundant features—those containing repeated information or having weak correlation with the classification target. In network intrusion detection, for instance, experts may design dozens of traffic statistical features, many of which are highly correlated. In small-sample scenarios where the number of data points is fewer than the number of features, models may struggle to identify important features and could mistake random fluctuations in redundant features for attack patterns. This often leads to good performance on the training set but poor generalization to new data.

To overcome the limitations of manual feature engineering, a raw traffic representation method based on structured tensor encoding is proposed. This method avoids reliance on prior expert knowledge and automatically learns discriminative features directly from the original hexadecimal sequence of network traffic. The encoding process consists of three main steps:

(1) Mapping from Hexadecimal Bytes to Decimal Values

The payload of each data packet is treated as a continuous hexadecimal string. The encoding process extracts every two characters from the string to form a hexadecimal byte, which is then converted into integers in the range of 0–255. This mapping is defined by Equation (1):

(1) $$v_{i}=f\left(B_{i}\right)=int(B_{i},16),$$

Among them, B_i_ is the i-th hexadecimal byte, and int (B_i_,16) represents parsing a hexadecimal string of length 2 into an integer based on 16.

(2) Generation and Filling of Fixed Length Sequence

Deep learning models typically require inputs to have a unified dimension. Therefore, it is necessary to convert the variable-length numerical vector into a fixed length T. T is a preset hyperparameter that represents the maximum sequence length that the model can handle. For a vector V = [v_1_, v_2_,…, v_T_] containing M values:

If M > T, the vector is truncated from the beginning, retaining the first T values: V’ = [v_1_, v_2_,…, v_T_]. If M < T, zeros are filled at the end of the vector until its length is equal to T: V’ = [v_1_, v_2_,…, v_M_, 0, 0,…, 0]. The truncation and filling operations can be expressed uniformly as Equation (2):

(2) $$V^{\prime }\left[j\right]=\left\{\begin{array}{c} V\left[j\right]\;If\;j\;\leq \min(M,T)\\ 0\;\;\;\;\;\;\;\;\;\;\;\;\;\;\;If\;M<j\leq T \end{array}\right.\;\;\;For\;j=1,\;2,\ldots ,\;T,$$

(3) Construction of structured tensors

**Figure 4:**
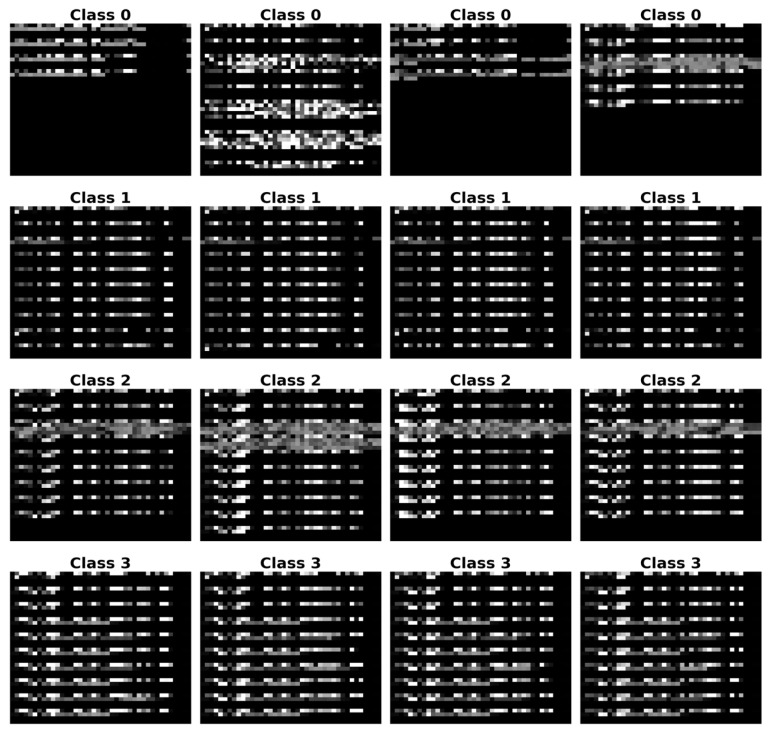
Grey-scale images of different types of traffic.

Reshape the fixed-length vector V’ into a three-dimensional tensor X∈R^B∗T∗C^, where B is the batch size, T is the sequence length, and C represents the number of feature channels (C = 1 in this work). Consequently, each raw data packet is encoded into a tensor of shape (T,1). For visual inspection of the encoded features, the structured tensor is transformed into a grayscale image. Specifically, the byte value sequence of each traffic sample is reshaped into a 64 × 64 two-dimensional matrix, where the grayscale value of each pixel corresponds to the normalized value of its respective byte. Figure 4 presents grayscale image samples from different categories, revealing distinct visual textures between normal traffic and various types of attack traffic.

In the model input stage, these 64 × 64 grayscale images are uniformly resized to 40 × 40 pixels. The scaling improves computational efficiency while preserving key features and the resulting single-channel image serves as the direct input to the subsequent convolutional neural network components, completing an end-to-end processing pipeline from raw data to classification results. The choice of the fixed sequence length T and the final image size is based on the following rationale. An analysis of packet length distribution in the CICIDS2017 dataset shows that the payload of the vast majority of packets is under 1600 bytes. Representing this as a 40 × 40 image (1600 pixels) ensures coverage of most actionable information in a compact 2D representation. This size represents a practical trade-off between computational efficiency and feature resolution: it provides sufficient spatial dimensions for the convolutional networks to learn both local and global patterns, yet keeps the per-sample input size manageable for efficient training of the subsequent CNN-LSTM model. Furthermore, an input size of 40 × 40 (or a similar power-of-two dimension, like 32 × 32 or 64 × 64) is a standard and widely validated choice in image classification tasks. Therefore, instead of conducting an exhaustive grid search over T and image size, we adopted this efficient and well-justified setting based on the aforementioned principles of data coverage, efficiency, and common practice.

### 3.2. Hierarchical Deep Learning Model

The model input is a 40 × 40 single-channel grayscale image, obtained via preprocessing and structured tensor encoding of the original network traffic. The CNN module comprises two convolutional layers with corresponding pooling layers, utilizing kernels of different sizes to capture local patterns. The first convolutional layer employs 32 filters of size 5 × 5 with a ReLU activation function and is followed by a 2 × 2 max pooling layer. The second convolutional layer employs 64 filters of size 3 × 3, also with ReLU activation, and is followed by another 2 × 2 max-pooling layer. After these two pooling stages, the feature map size is reduced to 8 × 8 with 64 channels. Subsequently, these feature maps are flattened into a 4096-dimensional vector and passed into a fully connected layer containing 1600 neurons for high-level feature integration. A dropout operation is applied after this layer to prevent overfitting. This section contains two convolutional layers and corresponding pooling layers, using convolution kernels of different sizes to capture local patterns. The convolution operation follows Equation (3), where *c_i,j_* are the pixel values of the new features generated by convolution at position (i, j), *δ_r_* is the activation function ReLU, and u and v represent the position indices of the convolution kernel in the vertical and horizontal directions, height and width dimensions, respectively, with values ranging from 0 to k − 1. *α_u_*_,*v*_ are the weights of the convolution kernel at position (*u*, *v*); *X_i_*_+*u*,*j*+*v*_ are the pixel values of the corresponding positions in the input image, and b is the bias term. The size variation of the feature map follows Equations (4) and (5), where inset and oversize represent the input and output sizes, respectively, k is the size of the convolution kernel or pooling kernel, s is the step size, and p is the fill size.

(3) $$c_{i,j}=\delta_{r}(\sum_{u=0}^{k-1}\sum_{v=0}^{k-1}\alpha_{u,v}\cdot X_{i+u,j+v}+b),$$

(4) $$outsize=\frac{insize-k+2p}{s}+1,$$

(5) $$outsize=\frac{insize-k}{s}+1,$$

After flattening, the feature maps are transformed into a feature vector by a fully connected layer. This vector is then reshaped into a sequence and fed into a two-layer LSTM network to capture temporal dependencies in the traffic data. Each LSTM layer contains 256 hidden units. The gating mechanism (forget gate, input gate, output gate) uses the sigmoid activation function, while the candidate values for updating the cell state are generated using the tanh activation function. This design enables the model to learn temporal dependencies and capture dynamic behavioral patterns across packets, with the gating mechanism effectively managing long-term information flow and mitigating the vanishing gradient problem. The LSTM unit effectively manages long-term information flow through its gating mechanism, avoiding the problem of gradient vanishing. In the gate control mechanism of LSTM, as shown in Equation (6), in the calculation of the forget gate, f_t_ represents the output of the forget gate at the current time, σ is the sigmoid function, *W_f_* is the weight matrix of the forget gate, *h_t_*_−1_ is the hidden state at the previous time, *x_t_* is the current input, and b_f_ is the bias term. Similarly, the input and output gates also achieve precise control over the information flow through corresponding weight matrices and activation functions.

(6) $$f_{t}=\sigma (W_{f}\cdot \left[h_{t-1},x_{t}\right]+b_{f}),$$

**Figure 5:**
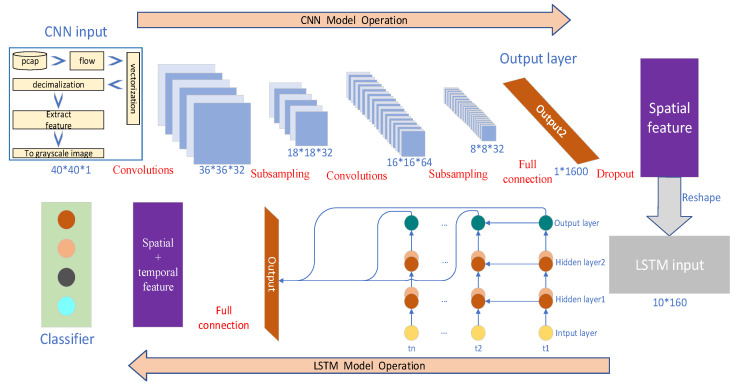
Flowchart of the hierarchical deep learning model.

The final model outputs a normalized probability distribution through the Softmax classifier, performing multi-class classification of network traffic. The entire network is trained end-to-end with parameters optimized using the Adam optimizer and a categorical cross-entropy loss function. This hierarchical structure leverages the respective strengths of CNN for spatial feature extraction and LSTM for temporal modeling, achieving a comprehensive learning of network traffic characteristics. The flowchart in Figure 5 visually illustrates the connection and information flow between modules, facilitating comprehension of the model’s overall workflow.

Having established the model infrastructure, attention must now turn to its security in adversarial environments, as the well-documented vulnerability of deep neural networks to adversarial attacks cannot be overlooked.

### 3.3. Multi-Strategy Adversarial Training Method

To address the vulnerability of deep learning models to adversarial attacks, this section proposes an efficient adversarial training method that integrates multiple attack strategies [34]. This method mixes adversarial samples in a phased, dynamically adjusted manner, aiming to achieve broad generalization robustness at a computational cost comparable to single-attack training.

Traditional adversarial training often relies on a single attack method, resulting in a narrow defense range. Simply mixing all attack samples, however, would incur significant computational overhead. To this end, we design a phased training framework. Its core idea is to first introduce strong, efficiently generated attack samples (e.g., FGSM and its multi-step variant, PGD) in the early training stage to rapidly establish a baseline of robustness. As training progresses, more refined, minimally-perturbing attacks (e.g., DeepFool) are gradually introduced to diversify the perturbation types the model encounters, forcing it to learn more discriminative feature boundaries. In the final stage, training focuses on a mixture of strong and refined attacks to further consolidate and sharpen the model’s defenses. Throughout the process, the mix of different attack samples is dynamically configured based on their computational costs and training utility.

This method integrates three typical white-box attack algorithms to generate adversarial samples. The FGSM method generates adversarial samples through single-step gradient updates, and its perturbation generation follows Equation (7):

(7) $$x^{\prime }=x+\varepsilon \cdot sign(\nabla_{x}J(\theta ,x,y)),$$

Among them, *ε* is the disturbance amplitude constraint, ∇*_x_J* represents the gradient of the loss function with respect to the input x, and sign ( ) is the sign function [7].

The PGD method, as a multi-step iterative extension of FGSM, generates stronger attack samples through the iterative process shown in Equation (8):

(8) $$x^{t+1}={Proj}_{B_{\varepsilon }\left(x\right)}(x^{t}+\alpha \cdot sign(\nabla_{x}J(\theta ,x^{t},y))),$$

Among them, ${Proj}_{B_{\varepsilon }\left(x\right)}$ represents projecting the perturbation onto a norm sphere with a radius of *ε* centered on the original sample x, and α is the step size [28].

The DeepFool method is based on geometric principles to find the minimum perturbation and calculates the minimum perturbation required to push the sample across the classification boundary through iterative linear approximation, as shown in Equation (9):

(9) $$\delta_{i}=arg\begin{array}{c} min\\ \delta \end{array}{\left|\left|\delta \right|\right|}_{2}\;\;\;s.t.\;\;\;f_{k}\left(x_{i}+\delta \right)\geq f_{\hat{y}}(x_{i}+\delta )$$

Among them, *f_k_* is the discriminant function of the k-th class, and $\hat{y}$ is the current predicted class. Iteratively update the sample position x_i+1_ = *x_i_* + *δ_i_* until misclassification occurs or the maximum number of iterations is reached. DeepFool can achieve attacks with small perturbation amplitudes, revealing the geometric characteristics of decision boundaries in deep learning models [8].

To balance training efficiency and defense effectiveness, this method employs a dynamic strategy selection mechanism. This mechanism automatically switches training stages and adjusts the mix of adversarial samples based on training progress, eliminating the need for manual intervention. The design accounts for the computational costs of different attack methods: attack samples that are fast to generate (e.g., FGSM) constitute a higher proportion in the early stages to rapidly build basic robustness; those with higher computational costs (e.g., DeepFool) are introduced in a controlled manner in the later stages to avoid compromising training efficiency. This approach ensures the model is progressively exposed to a diverse set of attack samples while maintaining total training time within a practical limit.

The principal advantage of this method is its simulation of a model’s adaptation to an increasingly complex adversarial environment through a single, coherent training process, leading to systematic robustness improvement. Compared to traditional approaches that require separate or sequential training for each attack type, our method achieves broad defense coverage while significantly reducing computational overhead. All specific hyperparameters, including stage divisions, duration ratios, and precise adversarial sample mixing ratios, are detailed in the experimental setup section (see Section 4.1) to ensure full reproducibility.

## 4. Experiment and Results Analysis

### 4.1. Experimental Setup and Dataset

Experiments are conducted using the CICIDS2017 dataset as the evaluation benchmark. This dataset simulates a realistic network environment and contains various typical network threats. Data was collected over five consecutive days, providing a comprehensive record of mixed network activities involving both normal traffic and malicious attacks. The distribution of traffic types within the dataset reflects characteristics of real-world environments, such as a high proportion of benign traffic and port scanning attacks, ensuring the practicality and representativeness of the experimental results. For this study, valid traffic samples from the dataset are used. These samples are randomly split into training and test sets in an 8:2 ratio. This split ensures a robust evaluation of the model on unseen data. Given the dataset size and our focus on maximizing the training data for the primary robust model, we did not employ a separate hold-out validation set for early stopping. Instead, the model was trained for a fixed number of epochs (2000) as specified in Table 1, which was determined to be sufficient for convergence based on preliminary experiments where training loss plateaued. The final performance is reported solely on the held-out test set.

Data preprocessing encompasses two main steps: cleaning and standardization. During the cleaning phase, the validity of the hexadecimal-encoded packet format is verified, and invalid or corrupted data are removed. In the standardization stage, hexadecimal sequences are converted into decimal numerical vectors, which are then uniformly adjusted to a fixed length through zero-padding or truncation operations. The entire preprocessing pipeline avoids manual feature selection, strictly preserves the integrity of the raw data, and lays a reliable foundation for subsequent feature learning.

The normalized decimal vectors, after being reshaped into 40 × 40 pixel arrays, undergo standardization (Z-score normalization) before being fed into the model. This process ensures that each feature dimension (i.e., each pixel location across the dataset) has approximately zero mean and unit variance. The mean (*μ_train_*) and standard deviation (*σ_train_*) are computed exclusively from the training set. Each sample x is then transformed as follows:

(10) $$x_{standardized}=\frac{x-\mu_{train}}{\sigma_{train}+\delta }$$

where δ = 1 × 10^−8^ is a small constant added for numerical stability. All adversarial perturbation budgets (e.g., *ε* = 0.15 for FGSM and PGD) are defined and applied within this standardized input space. This scheme provides a consistent and interpretable scale for evaluating the strength of adversarial attacks and ensures that the perturbation magnitude is meaningful relative to the unit variance of the feature distribution.

The experiment employs the hierarchical deep learning model based on structured tensor encoding described earlier. The input data is a 40 × 40 grayscale image, obtained by encoding and resizing the original flow data. The key hyperparameters and training configurations are summarized in Table 1. The specific reasons for choosing the current training stage and the proportion of training samples are explained in the experiment in Appendix A.

All experiments were conducted on a consistent hardware and software platform. The hardware configuration consisted of a personal computer with an Intel Core i5 processor, 16 GB of RAM, and an NVIDIA GeForce GTX 1650 GPU for accelerated model training. The software environment included the Windows 10 operating system, Python 3.10 as the programming language, the TensorFlow 2.13.0 deep learning framework, and the CUDA 11.6 toolkit for GPU acceleration. Key supporting libraries included NumPy and Pandas. Model performance was evaluated using four standard metrics: Accuracy, Precision, Recall, and F1-score. Accuracy measures overall classification correctness. Precision focuses on the correctness of positive predictions, while Recall reflects the model’s ability to identify all relevant positive cases. The F1-score provides a harmonic mean of Precision and Recall, offering a single metric for balanced assessment. These metrics, derived from the confusion matrix, comprehensively capture model performance across different scenarios and are particularly suitable for evaluating network traffic data with inherent class imbalances.

### 4.2. Comparison of Basic Detection Performance

The detection performance of the proposed method was systematically evaluated under normal network conditions, with tests conducted separately for multi-class and binary classification tasks. The multi-class task involves distinguishing specific attack types, whereas the binary task simply classifies traffic as benign or malicious. The corresponding performance metrics on the test set are presented in Table 2.

**Figure 6:**
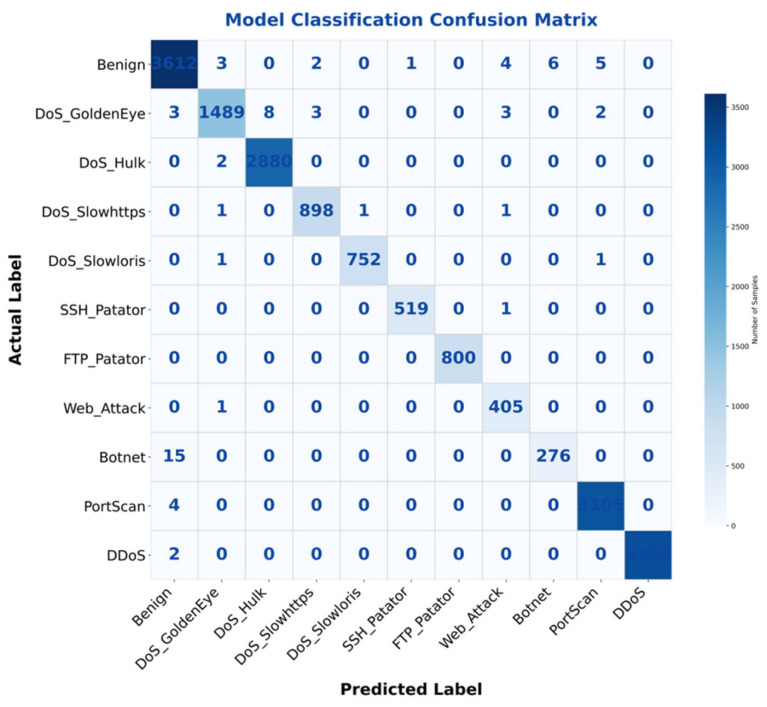
Confusion matrix for multi-class tasks.

Figure 6 shows the confusion matrix of the multi-class task. The strong diagonal concentration indicates high per-class recognition accuracy, while the lighter off-diagonal entries reflect minimal misclassification between categories.

**Figure 7:**
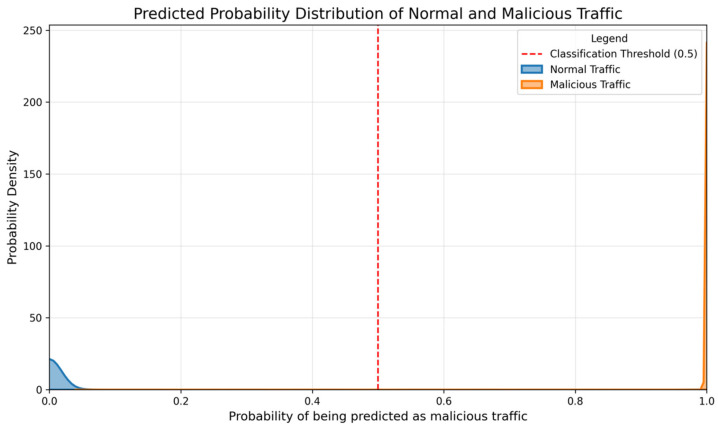
Probability distribution of binary classification task output.

For the binary classification task, Figure 7 displays the model’s output probability distribution. The probabilities for normal and malicious traffic are well separated with negligible overlap, demonstrating strong discriminative ability. The predictions cluster near the extremes (0 or 1), with almost no samples in the ambiguous middle range or immediately around the 0.5 decision threshold, indicating that classification errors are highly unlikely.

This study selected Random Forest, Support Vector Machine (SVM), and XGBoost as core baselines for comparison, based on three key considerations. First, representativeness: these models are long-established and widely adopted performance benchmarks in intrusion detection, enabling a direct performance comparison within a well-established evaluation framework. Second, paradigm contrast: a core innovation of our work is to circumvent the information loss inherent in manual feature engineering. The aforementioned classic models are prime representatives of the traditional “manual feature engineering + classifier” paradigm. Comparing against them directly validates the superiority of the proposed end-to-end learning paradigm from structured tensors. Third, practical relevance: owing to their good interpretability and computational efficiency, these models remain prevalent in deployed intrusion detection systems; comparison with them helps assess the practical utility of our method.

Among the traditional machine learning models tested, Random Forest achieved the best accuracy at 93.1%, while SVM and XGBoost reached 84.7% and 84.5%, respectively. All traditional models yielded lower F1-scores than our proposed method, indicating that the deep learning approach based on structured tensor encoding holds a significant advantage in feature learning. These experimental results are summarized in Table 3.

### 4.3. Vulnerability Analysis Under Adversarial Attacks

We evaluate the robustness of the model against adversarial attacks without any prior adversarial training. Three typical white-box attack methods were employed: FGSM, PGD, and DeepFool. The attack parameters were configured as follows for FGSM: the perturbation amplitude was set to *ε* = 0.15; for PGD, we used 10 iterations with a step size α = 0.03 and the same *ε* = 0.15; for DeepFool, the maximum number of iterations was set to 10.

**Figure 8:**
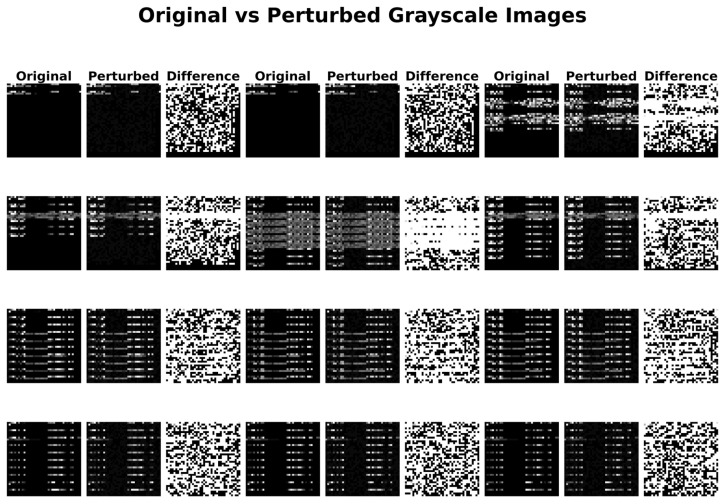
Comparison of grayscale images of data before and after attack.

To visually demonstrate the impact of adversarial perturbation, Figure 8 compares the original traffic data with FGSM adversarial samples using a series of grayscale images. The figure is organized into four rows, each showing a randomly selected sample from three typical traffic classes. Each row contains three columns: the original sample, its FGSM-attacked counterpart, and the difference between them. Visually, the adversarial perturbations are subtle and difficult for the human eye to discern, yet they are sufficient to cause significant changes in the model’s predictions.

The performance of the undefended model under different adversarial attacks is summarized in Table 4. Under baseline conditions, the model achieved an accuracy of 99.6% on benign test samples. When subjected to adversarial attacks, its performance dropped sharply. The FGSM attack was particularly effective, reducing accuracy to 24.4%—a drop of 75.2%. DeepFool also poses a serious threat, driving accuracy down to 29.9%. Under the current parameter settings, the PGD attacks had a more limited impact, with accuracy falling to 63.1%.

The performance variations under different attack methods reveal the model’s sensitivity to adversarial perturbations. Notably, FGSM and DeepFool cause substantially more severe degradation than PGD. The relatively limited impact of the PGD attack under the current setting may be attributed to two factors. First, as an iterative method, PGD’s final perturbation is constrained by both the initialization (typically the original sample) and the projection radius ε. With a limited iteration count (10) and the same ε as FGSM, its perturbative capability may not be fully realized. Second, the decision boundaries of an undefended model may be vulnerable in certain directions, which FGSM and DeepFool exploit more directly.

These results demonstrate that even a high-accuracy traffic classification model based on structured tensor encoding exhibits critical security vulnerabilities under adversarial attacks. The model is highly sensitive to small input disturbances and can be easily deceived by carefully crafted adversarial samples, despite its strong performance in a benign environment. This finding underscores the necessity of explicitly considering and enhancing adversarial robustness when deploying deep learning models in security-critical applications.

### 4.4. Enhancement of Robustness After Adversarial Training

To enhance the model’s defensive capabilities, it was strengthened using the proposed multi-strategy adversarial training. After training, the model’s robustness was evaluated against the same three white-box attack methods (FGSM, PGD, and DeepFool), with attack parameters consistent with the previous vulnerability analysis.

**Figure 9:**
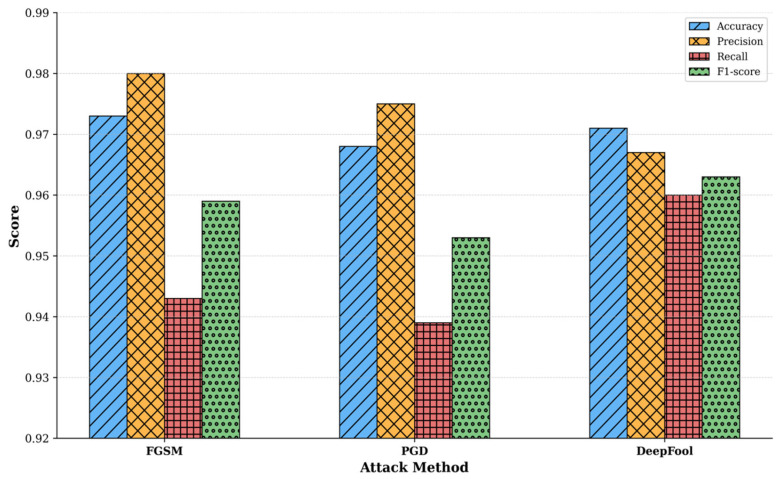
Defense effect of multi-strategy adversarial training.

As shown in Table 5 and Figure 9, the model achieves strong defense after multi-strategy adversarial training with a classification accuracy of 97.3% under FGSM attacks; 96.8% under PGD attacks, and 97.1% under DeepFool attacks. Compared to the undefended model, adversarial training yields significant improvements across all evaluation metrics. Notably, the model’s performance remains well-balanced against different attack types, with variations of less than 0.5% between them, indicating broad and consistent defensive coverage.

The simultaneous improvement in both accuracy and recall indicates that the model effectively identifies attacks while controlling the false positive rate. The balanced and high F1-scores further confirm the model’s reliability for practical deployment. Multi-strategy adversarial training enables the model to learn more generalized feature representations. Its maintained accuracy under diverse adversarial perturbations suggests that its decision boundaries have become smoother and more robust. This enhancement not only improves the model’s defense against the known attack types used in training but also strengthens the model’s potential to generalize against unknown attack variants. The experimental results demonstrate that the proposed multi-strategy adversarial training method effectively improves the model’s security and reliability in adversarial environments.

### 4.5. Analysis of the Balance Between Training Efficiency and Performance

Balancing training efficiency with performance while enhancing model robustness is crucial for practical deployment. This section compares the comprehensive performance and cost of two approaches: multi-strategy ensemble training (our proposed method) and multiple sequential training. The latter sequentially trains the model using one type of adversarial sample at a time—first with FGSM samples, then PGD, and finally DeepFool. The performance and efficiency data for both methods are compared in Table 6. The sequential method achieved marginally higher accuracy in certain scenarios, 97.9% against FGSM and 98.6% against DeepFool attack, but only 96.6% against PGD. In contrast, multi-strategy ensemble training exhibits more balanced performance across all attack types, with accuracies of 97.3%, 96.8%, and 97.1% against FGSM, PGD, and DeepFool, respectively.

The training time data shows that multiple sequential training takes 3185 s, which is more than three times the 1031 s required for multi-strategy ensemble training. This significant difference is due to the fact that multiple sequential training requires three complete training cycles, while multi-strategy ensemble training only requires a single training process. Multi-strategy ensemble training reduced the total training time by approximately 67.6%, demonstrating significant efficiency advantages.

The performance balance analysis further evaluated the impact of adversarial training on the normal classification ability of the model. As shown in Table 7, the model trained through multi-strategy adversarial training maintained excellent classification performance on normal samples, with an accuracy rate of 98.8%, with accuracy rate, recall rate, and F1 score all reaching 99.6%. This result indicates that adversarial training improves model security without compromising its ability to recognize normal traffic.

A comprehensive analysis reveals that multi-strategy ensemble training achieves a substantial gain in efficiency with only a minimal compromise in performance. This method delivers defense capabilities comparable to multiple sequential training in most test scenarios while dramatically cutting the training time. This efficiency advantage renders multi-strategy ensemble training particularly suitable for real-world applications that demand frequent and rapid model updates, offering a practical and efficient pathway toward deploying robust network intrusion detection systems.

## 5. Conclusions and Prospects

### 5.1. Research Summary

The experimental results demonstrate that the STS-AT framework achieves multiple key advances on the CICIDS2017 dataset. First, it reaches 99.6% accuracy in normal traffic classification, confirming the effectiveness of structured tensor encoding. Furthermore, multi-strategy adversarial training increases the model’s defense accuracy against FGSM, PGD, and DeepFool attacks from as low as 24.4% to over 96.8%, markedly enhancing robustness. Finally, the training strategy reduces total training time by approximately 67.6%, underscoring its efficiency. This work presents a complete STS-AT solution to core challenges in network intrusion detection. The structured tensor encoding enables direct feature learning from raw network traffic, avoiding the information loss typical of manual feature engineering. The designed hierarchical deep learning model integrates convolutional and long short-term memory networks to effectively capture the spatial characteristics and temporal dependency in traffic. To counter adversarial vulnerabilities, the proposed multi-strategy adversarial training introduces diverse adversarial samples in stages, significantly improving the model’s generalization robustness.

### 5.2. Comparison with Previous Work

The experimental results of this work should be situated within the broader research landscape. First, in comparison with classical machine learning methods reliant on manual feature engineering, the framework delivers superior classification performance. This directly validates the effectiveness of structured tensor encoding as an end-to-end learning paradigm for avoiding information loss and mining discriminative features. More importantly, the STS-AT framework demonstrates distinct advantages over existing adversarial training approaches. While traditional single-attack training yields a narrow defense range, naively integrating all attacks incurs prohibitive computational costs. In contrast, the proposed multi-stage adversarial training strategy intelligently schedules diverse attack samples (e.g., FGSM, PGD, and DeepFool) within a single training cycle. This achieves balanced, high-performance defense across multiple attack types while reducing training time by approximately 67.6%. This represents a substantive advance in addressing the key challenge of balancing robustness breadth with training efficiency.

### 5.3. Limitations and Future Prospects

The findings of this study are subject to several limitations that point to clear directions for future work. First, experimental validation relies primarily on the CICIDS2017 dataset. While representative, the model’s generalization requires further testing on more diverse network traffic containing newer attack variants. Second, the current framework involves inherent trade-offs. Structured tensor encoding depends on fixed-length truncation or padding, which prioritizes processing efficiency but may not optimally preserve discriminative information in unusually long or short packets. Furthermore, although multi-strategy adversarial training improves efficiency, it still incurs overhead from the dynamic generation of adversarial samples during training. Third, the baseline comparison focuses on classical machine learning models; a systematic comparison with state-of-the-art deep learning-based intrusion detection methods is needed to precisely position our contribution. Fourth, the current defense scope is limited to white-box gradient-based attacks. Evaluation against more covert threats—such as black-box, physical-world, or adversarial patch attacks—remains for future work. To address these limitations, future research can explore adaptive-length sequence modeling and more efficient adversarial robustness techniques to better balance performance, robustness, and cost. The architecture could also be enhanced by integrating attention or Transformer modules for improved spatiotemporal feature fusion. Finally, adapting the framework for resource-constrained edge computing scenarios (e.g., the IoT and vehicular networks) and exploring its integration with privacy-preserving paradigms like federated learning are promising directions for practical deployment.

## Figures and Tables

**Table 1:** Detailed parameter setting of model training and confrontation training.

Configuration Category	Parameter Name	Set Value	Remarks
Basic model training	Optimizer	Adam	
	Learning rate	0.0005	
	Batch size	128	
	Number of training rounds	2000	
	Loss function	Cross Entropy	
Adversarial sample generation	FGSM	*ε* = 0.15	
	PGD	*ε* = 0.15	
		Iteration steps = 10	
		Single step size α = 0.01	
	DeepFool	Maximum number of iterations = 2	
Multi-strategy adversarial training	Phase 1 adversarial sample ratio	FGSM:PGD = 7:3	30% before training cycle
	Phase 2 adversarial sample ratio	FGSM:PGD:Deepfool = 5:3:2	40% during training cycle
	Phase 3 adversarial sample ratio	PGD:Deepfool = 1:1	30% after training cycle

**Table 2:** Basic Performance Indicators of the Model.

Task Type	Accuracy	Precision	Recall	F1-Score
Multi-class classification	99.6%	99.7%	99.9%	99.8%
Binary classification	99.7%	99.6%	99.9%	99.8%

**Table 3:** Performance comparison with traditional machine learning models.

Model Name	Accuracy	Precision	Recall	F1-Score
Random Forest	93.1%	95.9%	82.1%	84.1%
SVM	84.7%	83.0%	84.7%	83.4%
XGBoost	84.5%	77.7%	73.4%	74.0%
Our model	99.6%	99.7%	99.9%	99.8%

**Table 4:** Comparison of model accuracy under different attack methods.

Attack Method	Perturbation Parameter	Accuracy After Attack
FGSM	*ε* = 0.15	24.4%
PGD	*ε* = 0.15, Iterate 10 times	63.1%
DeepFool	Maximum 10 iterations	29.9%
No attack (baseline)		99.6%

**Table 5:** Model defense performance after multi-strategy adversarial training.

Attack Method	Accuracy	Precision	Recall	F1-Score
FGSM	97.3%	98.0%	94.3%	95.9%
PGD	96.8%	97.5%	93.9%	95.3%
DeepFool	97.1%	96.7%	96.0%	96.3%

**Table 6:** Performance and efficiency comparison of two training methods.

Training Method	Test Scene	Accuracy	Training Time (s)
Multi-strategy integrated training	FGSM	97.3%	1031
	PGD	96.8%	
	DeepFool	97.1%	
Multiple sequential training	FGSM	97.9%	3185
	PGD	96.6%	
	DeepFool	98.6%	

**Table 7:** Normal sample classification performance of the model after adversarial training.

Evaluation Metrics	Numerical Value
Accuracy	98.8%
Precision	96.6%
Recall	96.6%
F1-Score	96.6%

## Data Availability

The data presented in this study are openly available in the Canadian Institute for Cybersecurity (CIC) repository at https://www.unb.ca/cic/datasets/ids-2017.html (accessed on 10 March 2025).

## Appendix A

### Appendix A.1

This experiment evaluated the impact of four training stage partitioning strategies on model performance while keeping the adversarial sample mix within each stage fixed. Besides the original phased progressive strategy (stage durations: 30%, 40%, 30%), three comparison strategies were tested: a uniform mixing strategy (equal stage durations), a late–heavy strategy (durations: 20%, 30%, 50%), and an early–heavy strategy (durations: 50%, 30%, 20%). The results (see Table A1) show that the original strategy delivered the most balanced and high-level defense accuracy (>96.8% against FGSM, PGD, and DeepFool) while also achieving the best trade-off in training time (1031 s). In contrast, the alternative strategies exhibited either inferior performance against specific attacks or reduced training efficiency. These findings validate the rationale behind the original stage division.

**Table A1:** Comparison of defense performance of different training strategies.

Attack Method	Original Strategy	Uniform Mixing	Late–Heavy	Early–Heavy
FGSM	97.3%	97.7%	97.7%	97.5%
PGD	96.8%	97.1%	97.1%	96.7%
DeepFool	97.1%	96.8%	97.5%	96.1%
Training time(s)	1031	1054	1134	977

### Appendix A.2

This experiment fixed the training stage divisions and evaluated three variant configurations—Conservative–Asymptotic, Balanced–Mixed, and Aggressive–Complex—by systematically adjusting the adversarial sample mixing ratio within each stage (specific ratios are provided in Table A2). Results in Table A3 show that each configuration exhibits a distinct performance profile. For instance, the Conservative–Asymptotic variant achieves the best defense against FGSM and PGD attacks but shows weaker performance against DeepFool. The Balanced-Mixed variant performs optimally under DeepFool attack but yields only average defense against PGD. In contrast, the original ratio configuration does not attain the highest accuracy on any single attack type. However, it delivers the most balanced and stable defense across FGSM, PGD, and DeepFool, with accuracy consistently around 97% and minimal fluctuation. It also maintains reasonable training efficiency. This indicates that the original ratio design enables the model to learn generalizable robust features, achieving an optimal balance among defense breadth, stability, and training efficiency.

**Table A2:** Sample ratio configuration within the stage.

Configuration Type	Training Phase	FGSM Ratio	PGD Ratio	DeepFool Ratio
Original proportion	Phase One	70%	30%	0%
	Phase Two	50%	30%	20%
	Phase Three	0%	50%	50%
Conservative–Asymptote	Phase One	80%	20%	0%
	Phase Two	60%	25%	15%
	Phase Three	0%	60%	40%
Balanced–Mixed	Phase One	60%	30%	10%
	Phase Two	40%	30%	30%
	Phase Three	0%	40%	60%
Aggressive–Complex	Phase One	50%	40%	10%
	Phase Two	30%	40%	30%
	Phase Three	0%	30%	70%

**Table A3:** Performance comparison of samples with different proportions within the stage.

Attack Method	Original Proportion	Conservative–Asymptote	Balanced–Mixed	Aggressive–Complex
FGSM	97.3%	97.9%	97.6%	95.9%
PGD	96.8%	97.1%	96.6%	94.9%
DeepFool	97.1%	95.2%	98.1%	97.1%
Training time (s)	1031	978	1086	1142
